# Cardiac Mineralocorticoid Receptor and the Na^+^/H^+^ Exchanger: Spilling the Beans

**DOI:** 10.3389/fcvm.2020.614279

**Published:** 2021-01-20

**Authors:** Irene Lucía Ennis, Néstor Gustavo Pérez

**Affiliations:** Centro de Investigaciones Cardiovasculares “Dr. Horacio E. Cingolani”, Facultad de Ciencias Médicas de la Plata, Universidad Nacional de La Plata, La Plata, Argentina

**Keywords:** aldosterone, mineralocorticoid receptor (MR), myocardial stretch, heart failure, NHE1, oxidative stress

## Abstract

Current evidence reveals that cardiac mineralocorticoid receptor (MR) activation following myocardial stretch plays an important physiological role in adapting developed force to sudden changes in hemodynamic conditions. Its underlying mechanism involves a previously unknown nongenomic effect of the MR that triggers redox-mediated Na^+^/H^+^ exchanger (NHE1) activation, intracellular Na^+^ accumulation, and a consequent increase in Ca^2+^ transient amplitude through reverse Na^+^/Ca^2+^ exchange. However, clinical evidence assigns a detrimental role to MR activation in the pathogenesis of severe cardiac diseases such as congestive heart failure. This mini review is meant to present and briefly discuss some recent discoveries about locally triggered cardiac MR signals with the objective of shedding some light on its physiological but potentially pathological consequences in the heart.

## Introduction

**Figure 1:**
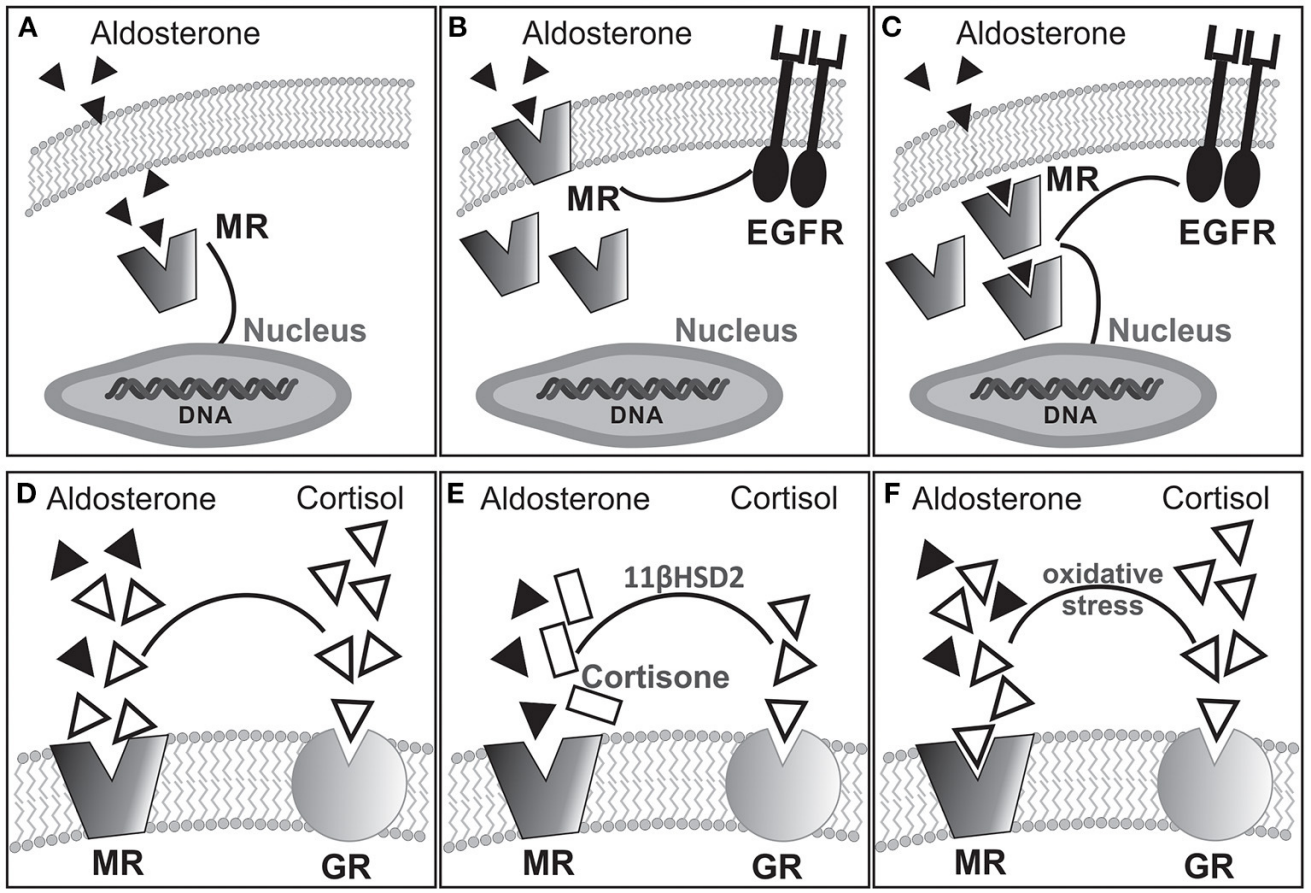
Hypothetical alternative pathways for MR activation-dependent effects: **(A)** classical cytosolic MR linked to genomic response; **(B)** membrane bound MR transactivating EGFR for nongenomic response coexisting with cytosolic MR; **(C)** cytosolic MR triggering both genomic and nongenomic responses. Experimental evidence suggests that rapid nongenomic effects would involve a subpopulation of MR located in the plasma membrane (1, 8, 9) as shown in **(B)**. Furthermore, as with other steroid hormone receptors, nongenomic MR signaling relies on crosstalk with other signaling cascades, among which EGFR transactivation seems to be of importance (4–6, 10). **(D–F)** Possible MR occupation by ALD or cortisol under different conditions. In the absence of 11βHSD2 **(D)**, cortisol occupies but does not activate the MR. The presence of 11βHSD2 converts cortisol into cortisone allowing ALD occupancy/activation of the MR **(E)**. Interestingly, pathologic oxidative stress facilitates occupancy/activation of the MR by cortisol **(F)**.

Mineralocorticoids are steroid hormones mainly involved in electrolyte/water balance regulation. Aldosterone (ALD) is the main hormone in this group. It is synthesized in the zona glomerulosa of the adrenal gland cortex by the action of P-450 ALD-synthase from 11-deoxycorticosterone through a process mainly controlled by angiotensin II (AngII) and potassium. The effects of ALD on extracellular fluid volume regulation and sodium/potassium homeostasis result from activation of cytosolic mineralocorticoid receptor (MR) on polarized epithelia of kidney distal nephron, salivary and sweat glands, and colon. Once activated, MR translocates to the nucleus and operates as a ligand-dependent transcription factor, mediating classic genomic effects. Interestingly, rapid nongenomic actions of ALD, probably linked to the plasma membrane MR subpopulation (1), have been demonstrated (2–5). As with other steroid hormone receptors, nongenomic MR signaling relies on crosstalk with other signaling cascades among which epidermal growth factor receptor (EGFR) transactivation plays a prominent role (4–6). Remarkably, constitutive colocalization of MR and EGFR at the plasma membrane was proved in a heterologous expression system (7). Figures 1A–C schematically summarizes hypothetical signaling pathways for MR activation–dependent effects.

It seems reasonable to imagine that the impact of ALD on cardiovascular systems may arise from its effects on electrolyte/water balance. However, the striking salutary effects of MR antagonism in heart failure (HF) patients (11–18) invite to consider that local MR actions may play a role in the progression of this pathology. Although the myocardium has no- or extremely low levels of ALD synthase and 11β-OHase, making mineralocorticoid synthesis very unlikely, hormone content in the heart tissue suggests that it could be synthesized and/or up-taken by cardiac cells (19, 20). Interestingly, significant amounts of ALD were detected in 30% of adrenalectomized rat hearts (20). This review was conceived to present/discuss evidence suggesting locally triggered cardiac MR signals with the aim of shedding light on its role in physiology and pathophysiology.

## The Mineralocorticoid Receptor

The MR is a member of the steroid/thyroid hormone receptor superfamily of ligand-inducible transcription factors, which also includes the glucocorticoid receptor (GR). Although increased ALD concentration constitutes the best-recognized stimulus for MR activation, it can also be stimulated under normal or even low ALD. Furthermore, it has high affinity for ALD and also for cortisol and 11-deoxycorticosterone (21). The latter is a relevant issue because the circulating glucocorticoid level is at least two orders of magnitude greater than ALD, determining that MRs are usually occupied although not activated by glucocorticoids (Figure 1D). In epithelial ALD-target cells, high expression of 11β-hydroxysteroid dehydrogenase (11βHSD2) facilitates ALD occupancy/activation of the MR by converting active glucocorticoids (cortisol) into receptor-inactive 11-keto analogs (cortisone, Figure 1E). In nonepithelial tissues, such as the myocardium, expression of 11βHSD2 is extremely low to prevent MR occupancy by cortisol, being the mechanism of MR activation by ALD certainly unknown. Interestingly, under pathologic conditions of increased oxidative stress, glucocorticoids can activate the MR (Figure 1F) (22). In this context, ALD-independent MR activation mechanisms might be considered: (1) glucocorticoid-mediated MR activation, especially under conditions of enhanced reactive oxygen species (ROS) (22), (2) ligand-independent (Rac1-dependent) MR activation (23), (3) direct MR phosphorylation independent of its own ligand as proposed for estrogen receptors (24), and (4) specific strain-induced conformational changes as proposed for AT1 receptor activation by mechanical stretch (25).

## The NHE1

The NHE1 is the predominant NHE isoform in cardiac tissue. This acid-extruding integral membrane protein mediates the electroneutral transport exchange of one intracellular H^+^ for one extracellular Na^+^, being the main responsibility of intracellular pH (pHi) regulation in the myocardium. It possesses high sensitivity to changes in pHi by allosterically sensing H^+^ and also to phosphorylation or interaction with associated proteins that modulate its activity (26). Its membrane domain (~500 amino acids) is related to ion transport, and a cytosolic tail (~315 amino acids) contains phosphorylation sites for different protein kinases (26). The NHE1 is constitutively phosphorylated in resting cells, a property that, in combination with its precise H^+^ sensing capability, guarantees its important housekeeping basal function. However, further phosphorylation may be induced by several stimuli, including factors involved in the development of severe cardiac diseases (26–28). For the objective of this review, it is important to highlight that ALD is able to stimulate the NHE1 through a nongenomic pathway as reported many years ago by Alzamora et al. (2) and Michea et al. (3) in different arteries. Interestingly, Alzamora et al. also show that inhibition of 11βHSD by carbenoxolone allowed cortisol to stimulate the NHE1. This important finding reveals that 11βHSD activity is also important in the context of rapid ALD actions, adding more interest to the regulation of NHE1 by mineralocorticoids/glucocorticoids, an issue that certainly deserves further investigation. In cardiac tissue, our own laboratory demonstrated that ALD triggers NHE1 stimulation also through a nongenomic pathway, an effect that requires EGFR transactivation (4, 5). Previous studies show that ALD upregulates NHE1 expression and function (27, 29, 30), an effect that promotes cell growth in neonatal ventricular cardiomyocytes (27). On the other hand, experiments by Fujisawa et al. (31) demonstrate a link between NHE1 activation and mineralocorticoid/salt-induced cardiac hypertrophy and fibrosis because both pathologic manifestations are prevented by specific exchanger inhibition. Contemporarily, experiments by Young and Funder (32) in the same model suggest that cardiac fibrosis may involve coronary vascular smooth muscle cell NHE1 activation.

In summary, two different scenarios involve NHE1 activity: Although basal NHE1 activity is crucial to maintaining cell homeostatic equilibrium, an enhanced exchanger activity would certainly lead to cardiac disease.

## Cardiac MR Activation by Stretch, NHE1 Activation, and Slow Force Response

**Figure 2:**
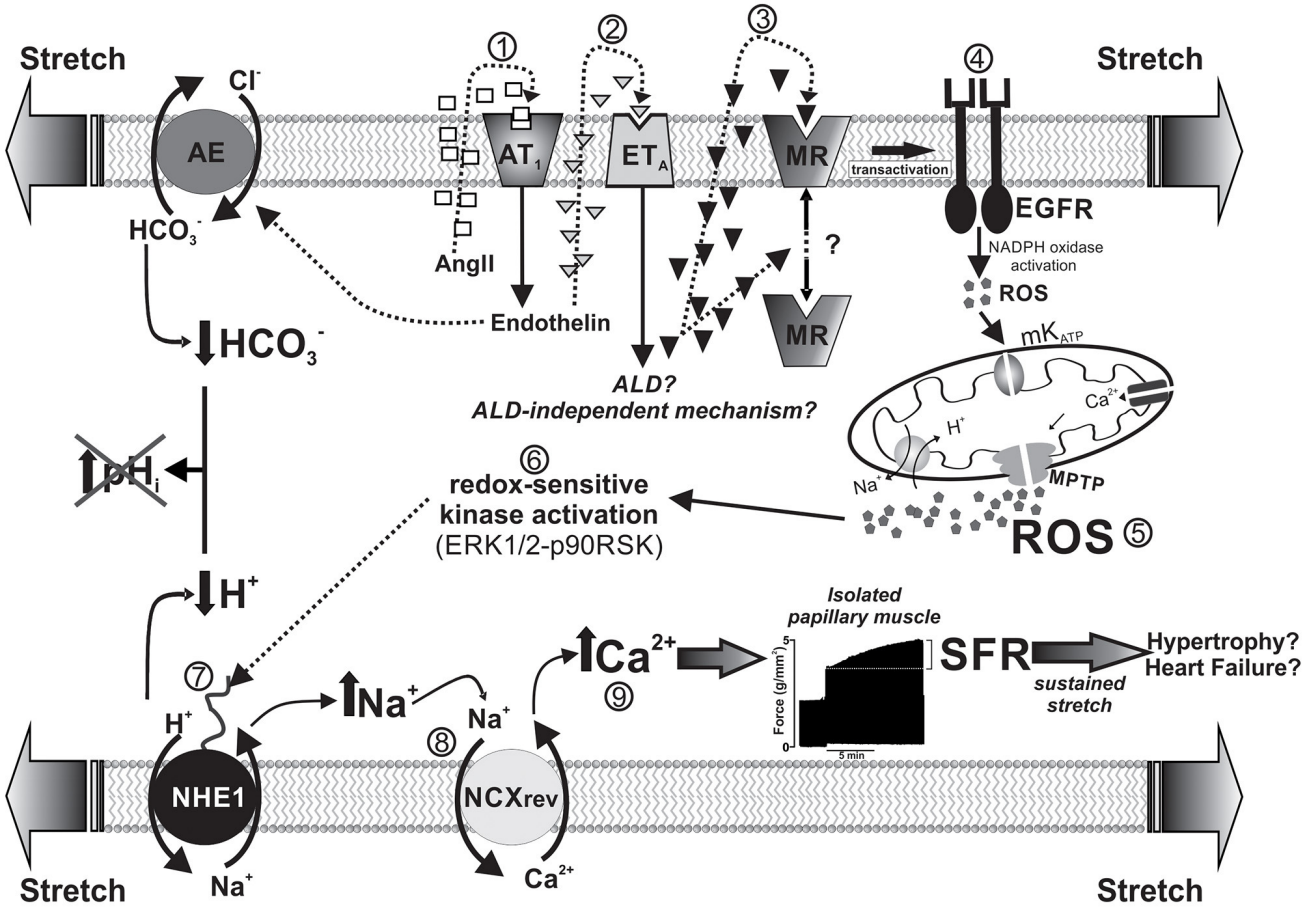
Schematized sequence of events triggered by myocardial stretch (from “1” to “9”) leading to the SFR and eventually to cardiac hypertrophy and failure. The mechanism begins with the activation of AngII (AT1) receptors by the mechanical stimulus and is followed by a sequential activation of endothelin, mineralocorticoid, and epidermal growth factor receptors (ET_A_, MR, and EGFR, respectively). Downstream of EGFR, a small amount of ROS triggers a greater production/release of ROS from the mitochondria, which, in turn, leads to NHE1 stimulation through redox-sensitive kinases. NHE1 activation increases intracellular Na^+^ and, thus, Ca^2+^ transient amplitude through reverse Na^+^/Ca^2+^ exchange (NCX_rev_), leading to the SFR (represented by a typical original force record from an isolated papillary muscle suddenly stretched). Interestingly, NHE1 activation does not modify intracellular pH because of the simultaneous activation of the acidifying Cl^−^/${\text{HCO}}_{3}^{-}$ anion exchanger (AE) by endothelin. The idea is that this sequence of events serves *in vivo* to adapt cardiac force to sudden changes in hemodynamic conditions but also may conceivably lead to cardiac hypertrophy and failure if the mechanical stimulus persist on time (e.g., under conditions of arterial hypertension).

Increased left ventricular end-diastolic volume caused either by increasing aortic resistance or venous return stretches the ventricular wall and immediately leads to a more powerful contraction. This adaptive response occurs in two consecutive phases: the immediate Frank-Starling mechanism attributed to enhanced myofilament Ca^2+^ responsiveness, followed by a gradual increase in force called “slow force response” to stretch (SFR), due to an increase in Ca^2+^ transient amplitude, and accounting for ~20% of total increase in force. *Ex vivo* studies in isolated papillary muscles isometrically contracting serve to describe a complex signaling cascade underlying the SFR, in which stretch-triggered NHE1 activation is crucial. The NHE1 could potentially increase force by two mechanisms: an increase in pHi that would increase myofilament Ca^2+^ sensitivity and/or an increase in intracellular Na^+^ leading to Ca^2+^ increase. We have described a mechanism that comprises a sequential activation of receptors (AT1, ET_A_, MR, and EGFR) leading to redox-mediated NHE1 stimulation, which increases intracellular Na^+^, and thus Ca^2+^ transient amplitude through reverse Na^+^/Ca^2+^ exchange. The scheme of Figure 2 summarizes this sequence. Interestingly, although activation of the NHE1 is critical, pHi does not play a role because of simultaneous activation of the acidifying Cl^−^/${\text{HCO}}_{3}^{-}$ anion exchanger by endothelin (33–35), which compensates the rise in pHi but not in Na^+^, leading to Ca^2+^ increase.

The demonstration that MR activation is crucial to increase mitochondrial ROS, leading to SFR development (4, 36, 37) appears to be particularly relevant given its potential clinical implications. Previous evidence guided us to explore the possible MR activation following stretch: (1) the recognized link between AT1 receptor activation and ALD (1, 38) and (2) the fact that EGFR transactivation [crucial for the SFR (39, 40)] can be triggered by MR activation (1, 7, 41). We have demonstrated cancellation of the SFR by MR blockade either pharmacologically (4) or by specific silencing of its expression (37) but not by glucocorticoid antagonism or protein synthesis inhibition (4). These results prove that activated MR is essential to modulate cardiac force response to stretch through a previously undescribed nongenomic effect that involves ROS-mediated NHE1 activation. Unfortunately, whether locally produced ALD participates in this mechanism remains unknown.

## Mineralocorticoid Receptor Activation and Heart Failure

HF is a chronic progressive condition in which the heart is unable to pump enough blood to meet organ/tissue requirements. It constitutes one of the most important global health problems. Current treatment is primarily based on inhibition of hormones, mainly AngII, ALD, and catecholamines. The pathophysiological role of ALD in cardiovascular disease has been classically attributed to sodium/water retention and hypertension due to MR activation in renal tubular cells. However, growing evidence suggests that local MR activation in the cardiovascular system may play a role. Although positive correlation between plasma ALD and cardiovascular damage has been described (42), complete inhibition of vascular angiotensin-converting enzyme in HF patients did not normalize plasma ALD (43). Furthermore, combining this treatment with AT1 antagonism only transiently reduced plasma ALD, suggesting the existence of AngII-independent production of ALD (44). This phenomenon called “ALD escape” provides a rationale to suggest direct MR inactivation instead of AngII blockade to improve HF treatment. In fact, several studies demonstrate striking beneficial effects of MR antagonism in HF patients (11–18). However, the mechanism underlying this action is still rather unknown.

The first MR antagonist accepted for clinical use ~60 years ago was spironolactone. Beyond some tolerability problems, it was approved for the Randomized Aldactone Evaluation Study (RALES) clinical trial in patients with severe HF (Class III-IV NYHA). This study was terminated prematurely due to an interim analysis revealing ~30% reduction in relative risk of death in spironolactone-treated patients together with an impressive reduction in hospitalization for cardiac reasons (11). Afterward, the more specific MR antagonist “eplerenone” was developed and clinically tested in the Eplerenone Post-Acute Myocardial Infarction Heart Failure Efficacy and Survival Study (EPHESUS), which recruited patients with acute myocardial infarction and left ventricular systolic dysfunction. The results showed ~15% reduction in all-cause mortality and ~21 % decrease in sudden cardiac death (12). The more recent Eplerenone in Mild Patients Hospitalization and Survival Study in Heart Failure (EMPHASIS-HF) was carried out on patients with less severe HF (class II-III NYHA) (13). Once again, the study was stopped early after a median follow-up of 21 months due to the remarkable reduction in the risk of cardiovascular death or hospitalization in the eplerenone arm, which was then extended to all patients.

A new generation of nonsteroid MR blockers with more selectively target benefits, reduced risks of altered receptor affinity and tissue tropism, and minimized side-effects such as hyperkalemia has been recently developed: finerenone, apararenone, and esaxerenone (18). Finerenone appears to be the most clinically advanced as evidenced by the minerAlocorticoid Receptor antagonist Tolerability Study-Heart Failure (ARTS-HF) study (15, 16). This phase IIb dose-finding study compared different dose groups of finerenone and eplerenone in HF patients with reduced ejection fraction and coexisting moderate chronic kidney disease and/or type-2 diabetes mellitus. The beneficial effects of this novel compound were certainly promising (16), but further studies are necessary to reach definitive conclusions. A large multicenter phase III trial recently approved (FINESSE-HF; EUCTR2015-002168-17-SE) will provide answers.

In contrast to the beneficial effects observed in all these studies, two clinical trials failed to show benefits after MR antagonism. In the Treatment of Preserved Cardiac Function Heart Failure with an Aldosterone Antagonist (TOPCAT) study, spironolactone did not improve clinical outcomes in patients with symptomatic HF with relatively preserved ejection fraction (≥45%) (45). In the Aldosterone Lethal effects Blockade in Acute myocardial infarction Treated with or without Reperfusion to improve Outcome and Survival at Six months follow-up (ALBATROSS) trial, a single intravenous bolus of potassium canrenoate followed by 6-months oral spironolactone failed to benefit patients admitted for myocardial infarction irrespective of the presence of HF or left ventricular dysfunction (46). These results clearly hamper any general conclusions about efficacy of MR antagonism in HF patients.

## Discussion

### Cardiac Mineralocorticoid Receptor Activation: From Physiology to Pathophysiology

As stated, the heart possesses a powerful intrinsic capacity to adjust cardiac output to abrupt changes in hemodynamic conditions. The stretch of ventricular wall is the mechanical stimulus that triggers such adaptation. The Frank-Starling law is perhaps the best-known mechanism to explain how force increases after stretch. However, the lesser known SFR constitutes an important additional adaptive mechanism that deserves special attention. Interestingly, critical events leading to the increase in Ca^2+^ transient amplitude that underlies the SFR (hormone release, oxidative stress, NHE1 hyperactivity) are also involved in the progression of severe cardiac pathologies such as hypertrophy and HF (26, 47, 48), whose trigger is wall stretch. Briefly, NHE1 hyperactivity caused by stretch-triggered mediators leads to intracellular Na^+^ and Ca^2+^ overload, calcineurin activation, and pathological cardiac hypertrophy. It is tempting to hypothesize that ventricular wall stretch may not only activate instant intrinsic heart mechanisms to adapt cardiac force to varying hemodynamic conditions, but also would represent an early step toward cardiac pathology if the mechanical stimulus persists over time. In this scenario, MR-triggered NHE1 activation after stretch would imply a double-edged sword with potentially important clinical implications.

### Certainties, Uncertainties, and Controversies

The important benefits of MR antagonists in different HF patient populations appear to be a certainty (11–18). However, their clinical use remains lower than expected. The reasons are not yet apparent but probably involve uncertainties arising from some clinical trials, together with poor knowledge about the exact mechanism of protection of these compounds. As described, three different clinical trials show irrefutable evidence about the outstanding beneficial effects of MR inactivation in HF patients under different clinical conditions: severe stage (RALES), post myocardial infarction with left ventricular systolic dysfunction (EPHESUS), or with mild symptoms (EMPHASIS-HF). However, the TOPCAT and ALBATROSS studies fail to support these findings, certainly challenging or at least limiting the possibility of reaching general conclusions about effectiveness of MR antagonism in HF. There is no conclusive explanation for these discrepancies. The heterogeneous population of ALBATROSS in terms of cardiac function/dysfunction appears to limit any profound analysis/comparison to other studies. However, it is worth noting that a possible reduction of mortality in the ST-segment elevation subgroup was reported (46), which may be due to prevention of adverse remodeling and/or potentially lethal arrhythmias. In TOPCAT, methodological uncertainties seem to weaken the analysis. Because the study enrolled patients from different countries, it is argued that heterogeneity of practice patterns and/or accuracy of diagnostic procedures to identify patients with relatively preserved ejection fraction would preclude reaching reliable results (49). Actually, beneficial effects in patients from American countries not observed in those from Russia and Georgia were reported (45). This encourages thinking that, if properly evaluated, MR antagonism would also improve outcomes even in these subpopulations (50).

Although described discrepancies invite caution, it seems possible to split conclusions. Although clinical evidence demonstrates benefits of MR inactivation in patients with severe and mild systolic HF, it is not clear whether it plays a role in patients with relatively preserved ejection fraction. Furthermore, MR antagonism seems to lack benefits when applied after myocardial infarction irrespective of ventricular function. Beyond these controversies, the mechanism by which MR antagonists provide protection in the first subgroup of patients are not completely understood. However, the demonstration from basic science studies that MR-NHE1 activation occurs early after acute myocardial stretch may shed some light. Further basic/clinical investigation is still necessary to reach definitive conclusions.

### Concluding Remarks

The striking benefits of MR antagonism to treat severe cardiac dysfunction support the notion that, under certain clinical conditions, MR activation is harmful. However, an important aspect to elucidate is the exact responsibility for this deleterious activation: ALD, physical deformation of MR, other ligands? No evidence assigns responsibility to ALD for MR activation despite demonstrated direct actions of ALD on the myocardium (5, 36, 51–53). Furthermore, MR holds equivalent high affinity for ALD and cortisol (21), but circulating levels of cortisol are greater (54). It is hard to conceive that ALD would have the chance of stimulating MR in the myocardium where low expression of 11βHSD2 could not prevent receptor occupancy by cortisol. Future research may unveil alternative explanations to support MR activation by ALD or even ALD-independent mechanisms such as those suggested in a previous section.

Another crucial issue to clarify is the exact mechanism of cardioprotection of MR antagonism. Although diuretic action of MR antagonists was initially considered, experimental evidence challenges this possibility. Interestingly, basic research findings allow the hypothesis that prevention of oxidative stress and, consequently, NHE1 hyperactivation may be crucial for their salutary effects in humans.

Finally, a critical challenge for the near future would be to design novel therapies to specifically antagonize myocardial MR activation. This procedure would avoid serious undesired side effects among which hyperkalemia should be remarked. Therefore, organ-specific MR antagonism appears to be the immediate next challenge for clinical practice in this field. In this regard, nonsteroidal MR antagonists and protein expression silencing strategy (as used experimentally) emerge as suitable alternatives.

## Author Contributions

ILE and NGP contributed equally to planning, writing, and critical reading of this manuscript. All authors contributed to the article and approved the submitted version.

## Conflict of Interest

The authors declare that the research was conducted in the absence of any commercial or financial relationships that could be construed as a potential conflict of interest.
